# Two new species of *Begonia* sect. *Coelocentrum*, *B. guixiensis* and *B. longa*, from Sino-Vietnamese limestone karsts

**DOI:** 10.1186/s40529-014-0052-8

**Published:** 2014-06-28

**Authors:** Ching-I Peng, Shin-Ming Ku, Hsun-An Yang, Wai-Chao Leong, Yan Liu, Tien Hiep Nguyen, Yoshiko Kono, Kuo-Fang Chung

**Affiliations:** 1 Herbarium (HAST), Biodiversity Research Center, Academia Sinica, Nangang 115, Taipei, Taiwan; 2 School of Forestry and Resource Conservation, National Taiwan University, Daan 106, Taipei, Taiwan; 3 Guangxi Institute of Botany, Guangxi Zhuang Autonomous Region and Chinese Academy of Sciences, Guilin, 541006 China; 4 Center for Plant Conservation (CPC), Vietnam Union of Science and Technology Associations, №25/32, lane 191, Lạc Long Quân Rd, Nghia Dô, Ha Noi, Cau Giay district Vietnam

**Keywords:** Begonia brevipedunculata, Begonia cylindrica, Begonia vietnamensis, Begoniaceae, China, Chromosome number, Guangxi, Vietnam

## Abstract

**Background:**

In our recent molecular phylogenetic study of Asian *Begonia*, two undescribed species, *B. guixiensis sp. ined.* (S. Guangxi, China) and *B. longa sp. ined.* (Vietnam), were sampled and placed within the strongly supported clade composed of *Begonia* sect. *Coelocentrum* and other co-distributed rhizomatous species in the Sino-Vietnamese limestone karsts. While *Begonia* sect. *Coelocentrum* has been recircumscribed based on the phylogenetic relationships, *B. guixiensis sp. ined.* and *B. longa sp. ined.* remain illegitimate names. In continuation of our studies in Asian *Begonia*, these two new species are described and illustrated.

**Results:**

*Begonia guixiensis* resembles *B. cylindrica* in the peltate, subcoriaceous leaves, differing by the shape of ovary/fruit and the type of placentation. In aspect, *B. longa* bears a superficial resemblance to *B. brevipedunculata* in leaf shape in particular, differing by many other features such as the long internodes, shorter petioles and smaller leaves, longer peduncles and 3-locular ovary. The chromosome number of both new species is determined as 2*n* = 30.

**Conclusion:**

A careful study of the literature, herbarium specimens and living plants, both in the wild and in cultivation in the experimental greenhouse, support the recognition of the two new species, which are described and illustrated herein.

**Electronic supplementary material:**

The online version of this article (doi:10.1186/s40529-014-0052-8) contains supplementary material, which is available to authorized users.

## Background

In East Asia, the vast picturesque limestone karsts striding across the Sino-Vietnamese border are a major biodiversity hotspot (Hou et al. [2010]; Xu [1995]), distinguished for a suite of species-rich and narrowly distributed herbaceous plant groups [e.g., *Aspidistra* Ker Gawl. (e.g., Lin et al. [2013]), *Begonia* L. (e.g., Averyanov and Nguyen [2012]; Peng et al. [2012]; Peng et al. [2013]), *Elatostema* J.R. Forst. and G. Forst. (e.g., Wei et al. [2013]), *Impatiens* L. (e.g., Zhang et al. [2014]), several genera of Gesneriaceae (e.g., Huang et al. [2011]; Xu et al. [2011], [2012a], [b], [2014]; Chung et al. [2013]), etc.] confined to caves, fissures, and crevices of limestone rocks (Chung et al. [2014]; Clements et al. [2006]). Amongst the various plants found in the caves or cave-like microhabitats of Sino-Vietnamese limestone karsts, *Begonia* sect. *Coelocentrum* Irmsch. are one of the most prominent groups, comprising ca. 55 rhizomatous species characterized by parietal placentation (Doorenbos et al. [1998]; Ku [1999]; Gu et al. [2007]; Liu et al. [2007]; Peng et al. [2007], [2008], [2013]; Averyanov and Nguyen [2012]; Ku et al. [2008]) which is rarely seen in other Asian *Begonia* (Thomas et al. [2011]).

**Figure 1:**
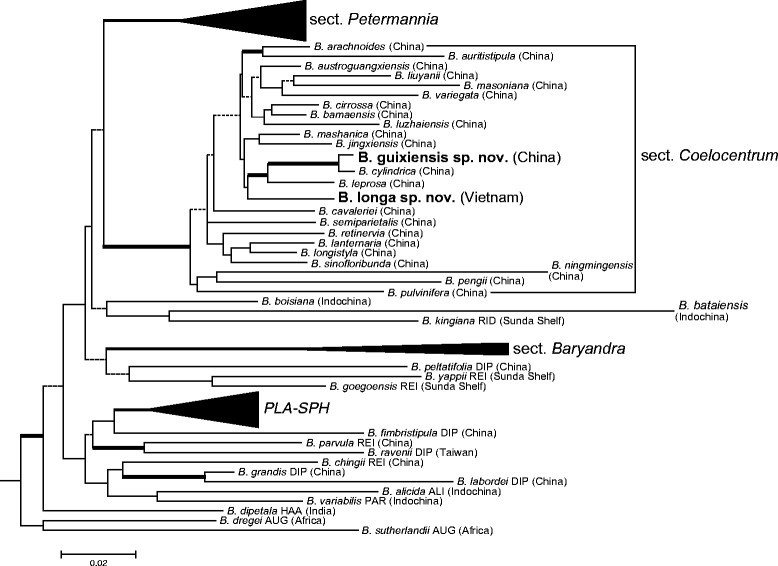
**Simplified phylogenetic tree based on the best-scoring maximum likelihood phylogram of Chung et al. (**[2014]**).** Clades of sect. *Petermannia,* sect. *Baryandra* and *PLA-SPH* (sections *Platycentrum* and *Sphenanthera*) are compressed for simplicity. Clade supports with less than 50%/0.5 in likelihood bootstrap (LB), parsimony bootstrap (PB), and posterior probability (PP) of Bayesian analyses are indicated by dashed branches, while thick branches denote those present in the strict consensus tree of maximum parsimony analysis and PP ≥0.95. Sectional abbreviations: ALI: *Alicida*, AUG: *Augustia*, DIP: *Diploclinium*, HAA: *Haagea*, PAR: *Parvibegonia*, REI: *Reichenheimia*, RID: *Ridleyella*, UA: unassigned.

Our recent molecular phylogenetic analysis (Chung et al. [2014]) demonstrates that *Begonia* sect. *Coelocentrum* is not monophyletic but instead dominates in a strongly supported clade that otherwise also includes *B. cavaleriei* H. Lév., *B. pulvinifera* C.-I Peng and Yan Liu, and *B. wangii* T.T. Yu of sect. *Diploclinium* (Lind.) A. DC., *B. cylindrica* D.R. Liang & X.X. Chen and *B. leprosa* Hance of sect. *Leprosae* (T.C. Ku) Y.M. Shui, and *B. sinofloribunda* Dorr of sect. *Petermannia* (Klotzsch) A. DC. Despite the disparity in placentation (vs. axile) and fruit types (cylindric and berry-like in sect. *Leprosae*), these six species are all rhizomatous and distributed exclusively in the Sino-Vietnamese limestone karsts. Given the strongly supported phylogenetic relationship, the presence of additional placentation and fruit types in the clade composed of species mainly with parietal placentation and dry capsule further attests the labile nature of ovary and fruit types for the infrageneric classification of *Begonia* highlighted by previous works (e.g., Tebbitt et al. [2006]; Thomas et al. [2011]). Because of the strongly supported phylogenetic relationship and apparent cohesiveness in terms of their perennation mode, geographic distribution, and ecological preference, Chung et al. ([2014]) expands the concept of *Begonia* sect. *Coelocentrum* to include the above-mentioned species. Meanwhile, in the phylogeny of Chung et al. ([2014]), two undescribed species, *B. guixiensis* sp. ined. and *B. longa* sp. ined., were sampled and grouped within the recircumscribed *Begonia* sect. *Coelocentrum* with strong support (Chung et al. [2014]; Figure 1). Detailed morphological description and cytological examination of the two new species are provided below.

## Methods

### Chromosome preparations

Somatic chromosomes of the two new species were examined using root tips. The methods followed Peng et al. ([2014a]). The classification of the chromosome complements based on the centromere position at mitotic metaphase described in Levan et al. ([1964]) was adopted. Voucher specimens (*Begonia guixiensis*: *Peng et al. 20310*; *B. longa*: *Peng et al. 20076*) were deposited in the Herbarium of the Biodiversity Research Center, Academia Sinica (HAST).

### Cryo scanning electron microscopy

The methods of sample preparation for cryo SEM described in Peng et al. ([2014a], [b]) were followed. Fresh leaves of *Begonia guixiensis* (*Peng et al. 20310*) and *B. longa* (*Peng et al. 20076*) were dissected and attached to a stub, frozen with liquid nitrogen slush, and then transferred to a sample preparation chamber at −160°C and etched for 15 min at −85°C. After etching, the temperature reached −130°C for sample fracturing and coating. After coating, the samples were transferred to the SEM chamber and observed at −160°C with a cryo scanning electron microscope (FEI Quanta 200 SEM/Quorum Cryo System PP2000TR FEI).

## Results and discussion

### Species description

**Figure 2:**
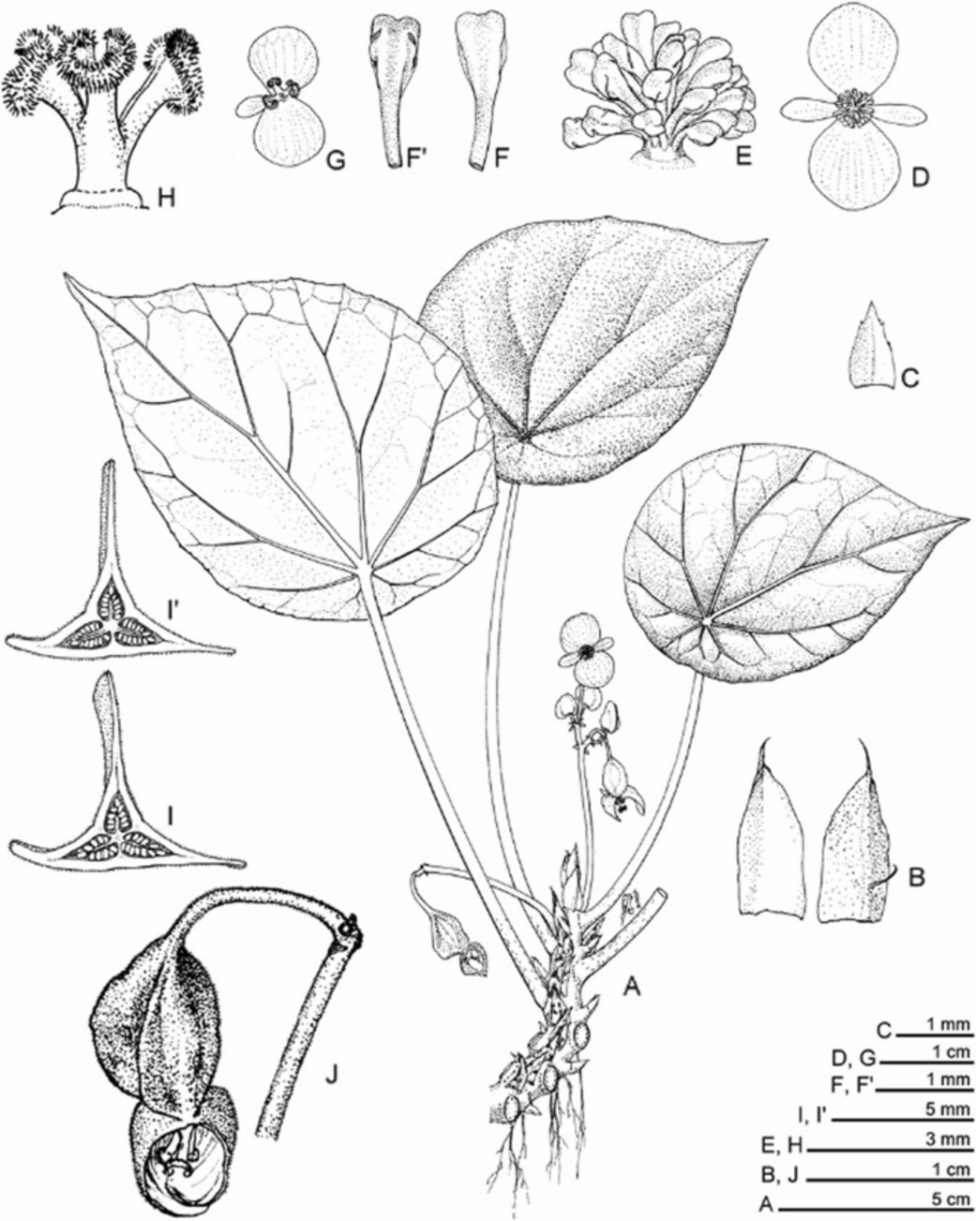
***Begonia guixiensis*** **Yan Liu, S.M. Ku & C.-I Peng. A**, Habit; **B**, Stipules; **C**, Bract; **D**, Staminate flower; **E**, Androecium; **F**, **F’**, Stamen; **G**, Carpellate flower; **H**, Style and stigmas; **I**, **I’**, Cross sections of ovary; **J**, 3-winged fruit. All from *C.-I Peng et al. 20310*. Line drawing by Ming-Chao Yu.

**Figure 3:**
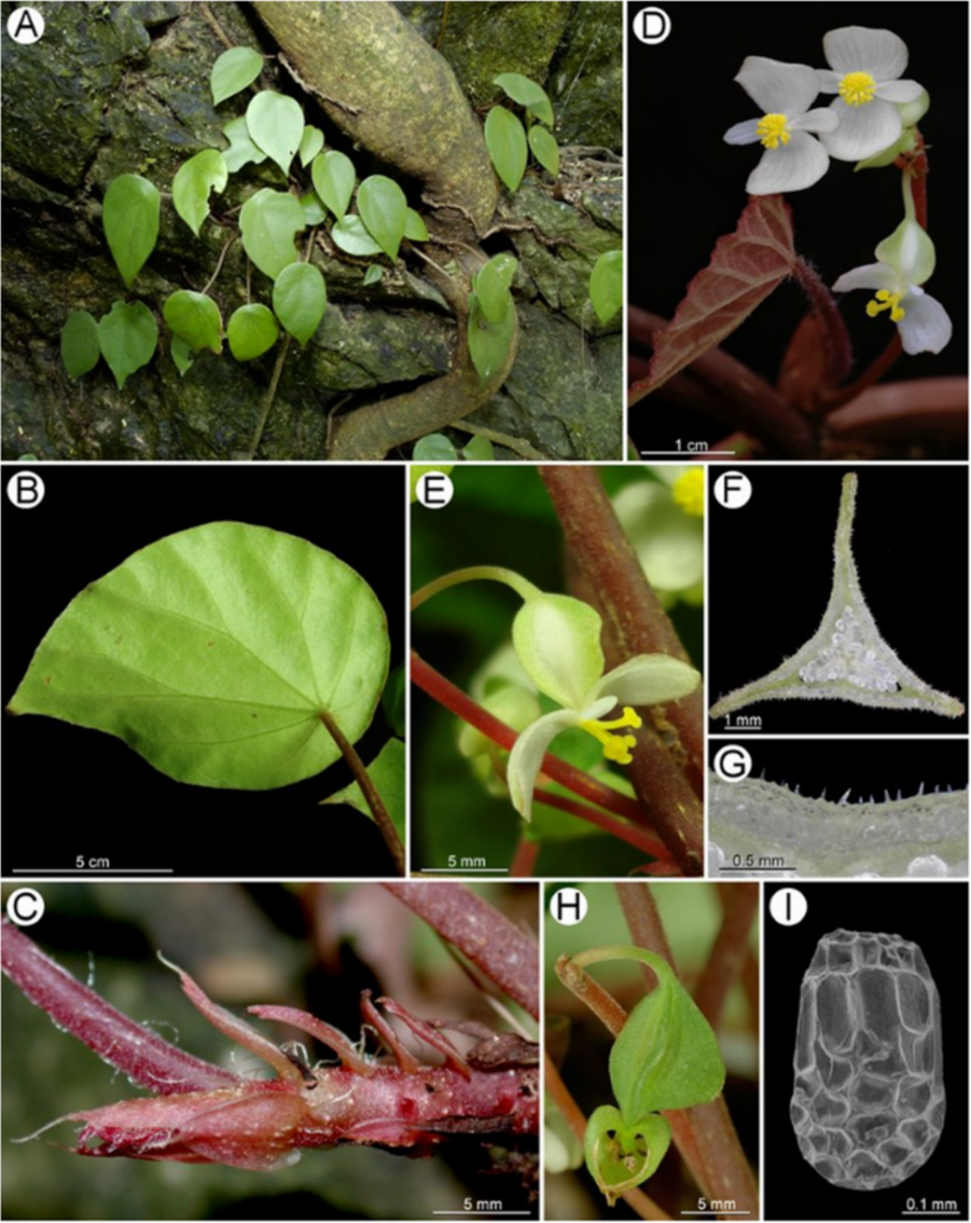
***Begonia guixiensis*** **Yan Liu, S.M. Ku & C.-I Peng. A**, Habit and habitat; **B**, Leaf, abaxial side; **C**, Rhizome and stipules; **D**, Flowers; **E**, Carpellate flower; **F**, Middle cross section of ovary; **G**, Unicellular hairs of ovary (minutely hispidulous); **H**, 3-winged fruit; **I**, Seed SEM micrograph. All from *C.-I Peng et al. 20310*.

1. ***Begonia guixiensis*** Yan Liu, S.M. Ku & C.-I Peng, sp. nov. (sect. *Coelocentrum*) —TYPE: CHINA, Guangxi Zhuang Autonomous Region, Chongzuo City, Mojian Tun, Longyana, on limestone rock face, N-facing, semishaded to heavily shaded, elev. ca. 150 m, 22°32′31”N, 107°29′23”E, locally frequent, most plants sterile, very few with dry fruits, 11 March 2005. Type specimens (in flower) pressed from plants brought back from the field and cultivated in the experimental greenhouse, Academia Sinica, Taiwan, 1 October 2013. *Ching-I Peng*, *Yan Liu*, *Shin-Ming Ku & Huan-Yu Chen 20310-A* (holotype: IBK; isotypes A, E, HAST, K, KUN, MO, PE). 桂西秋海棠 Figures 2 and 3.

Herbs monoecious, perennial, rhizomatous, lithophytic. *Rhizome* creeping, slender, to 30 cm or more, 0.3–0.7 cm across, internodes 0.2–0.7 cm long, glabrous or subglabrous. *Stipules* ovate-triangular, sometimes auriculate at base, 0.5–1 cm long, 0.3–0.7 cm wide, glabrous or with few hairs along midrib, margin entire and eciliate, apex aristate. *Leaves* simple, alternate; petiole 5–25 cm long, 3–5 mm across, densely and minutely hispidulous (unicellular trichomes ca. 0.1 mm long), sometimes sparsely pilose/villous when young (multicellular trichomes, 1–2 mm long); leaf blade green, peltate, petiole attachment displaced to one side, broadly ovate, 6.5–15 cm long, 4–10 cm wide, subcoriaceous, adaxially glabrous or nearly so, abaxially minutely hispidulous, margin entire or nearly so, apex acuminate or shortly so; basally 6- or 7-veined, tertiary veins obscure. *Inflorescences* axillary, 1–6 or more, arising directly from rhizome, dichasial cymes branched 2 or 3 times; 5–14-flowered; peduncle ascending, shorter than leaves, 2–6 cm long, 1.5–2 mm across, densely and minutely hispidulous; secondary peduncle usually short; bracts caducous, ovate-triangular to lanceolate, small, 0.6–2 mm long, 0.3–1.5 mm wide. *Staminate flowers:* bud suberect, pedicel 1–2 cm long, densely and minutely hispidulous; tepals 4, outer 2 suborbicular, 1–1.2 cm long, 0.8–1 cm wide, abaxially densely and minutely hispidulous, inner 2 narrowly obovate or oblanceolate, 0.7–0.8 cm long, 0.2–0.4 cm, glabrous; androecium actinomorphic, stamens 22–36, yellow, filaments fused at base, 1–1.5 mm long, anthers obovate-oblong, ca. 1 mm long. *Carpellate flowers:* pedicel 4–5 mm long; tepals 3, persistent in fruit, outer 2 suborbicular, 0.6–0.7 cm long, 0.7–0.8 cm wide, inner 1 narrowly obovate, 0.4–0.5 cm long, ca. 0.2 cm wide; ovary trigonous-ellipsoid, 0.8–1 cm long, ca. 3 mm wide, densely and minutely hispidulous (unicellular trichomes ca. 0.1 mm, whitish or reddish); placentation parietal on upper part of the ovary, placental branches gradually fused side by side below and assuming a 3-locular appearance; styles 3, fused 1/4 to 1/3 at base. *Fruit* capsule-like but indehiscent, trigonous-ellipsoid, 1–1.2 cm long, unequally or subequally 3-winged, abaxial wing subtriangular or lunate, 2–3.5 mm tall, peduncle recurved.

#### Ecology and distribution

**Figure 4:**
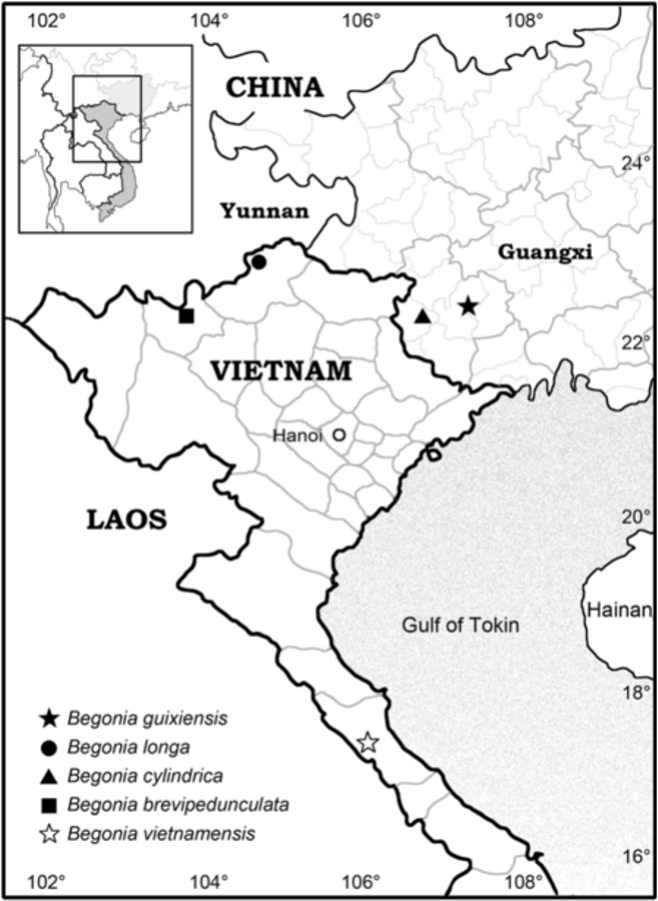
**Distribution map of** ***Begonia guixiensis*** **(**★**),** ***B. longa*** **(**●**),** ***B. cylindrica*** **(**▲**),** ***B. brevipedunculata*** **(**■**) and** ***B. vietnamensis*** **(**☆**).**

*Begonia guixiensis* is currently known only from Mojian Tun, Chongzhuo City, SW Guangxi, China (Figure 4). It grows on moist to dry surfaces of limestone hills, associated with *Alchornes* sp., *Ardisia* sp., *Asarum* sp., *Cleidion brevipetiolatum*, *Ficus tinctoria* subsp. *gibbosa*, *Mallotus yunnanensis*, *Miliusa chunii*, *Oplismenus* sp., *Pothos* sp., *Rhaphidophora hongkongensis*, *Sambucus* sp., *Smilax* sp. and *Tetrastigma* sp.

#### Phenology

Flowering July–October; fruiting August–November.

#### Etymology

The species epithet ‘guixiensis’ refers to Guangxi (acronym: ‘gui’) west (‘xi’), where the species is currently known.

#### Additional specimens examined

CHINA. Guangxi Zhuang Autonomous Region, Chongzuo Xian, Leizhou Xiang, Qubing Cun, Mojian Tun, Nongdan Nong, elev. ca. 160 m, in sparse forests on limestone hill slope, on rock face or rock seam, rare, flowers white, fruits green, 26 August 2004, *Comprehensive Expedition Team of Whiteheaded Langur Reserve B0570* (IBK). Guangxi Zhuang Autonomous Region, Chongzuo City, Mojian Tun, Longyana, on limestone rock face, N-facing, semishaded to heavily shaded, elev. ca. 150 m, 22°32′31”N, 107°29′23”E, locally frequent, most plants sterile, very few with dry fruits, 11 March 2005. *Ching-I Peng*, *Yan Liu*, *Shin-Ming Ku & Huan-Yu Chen 20310* (HAST)*.*

#### Leaf anatomy and vestiture

**Figure 5:**
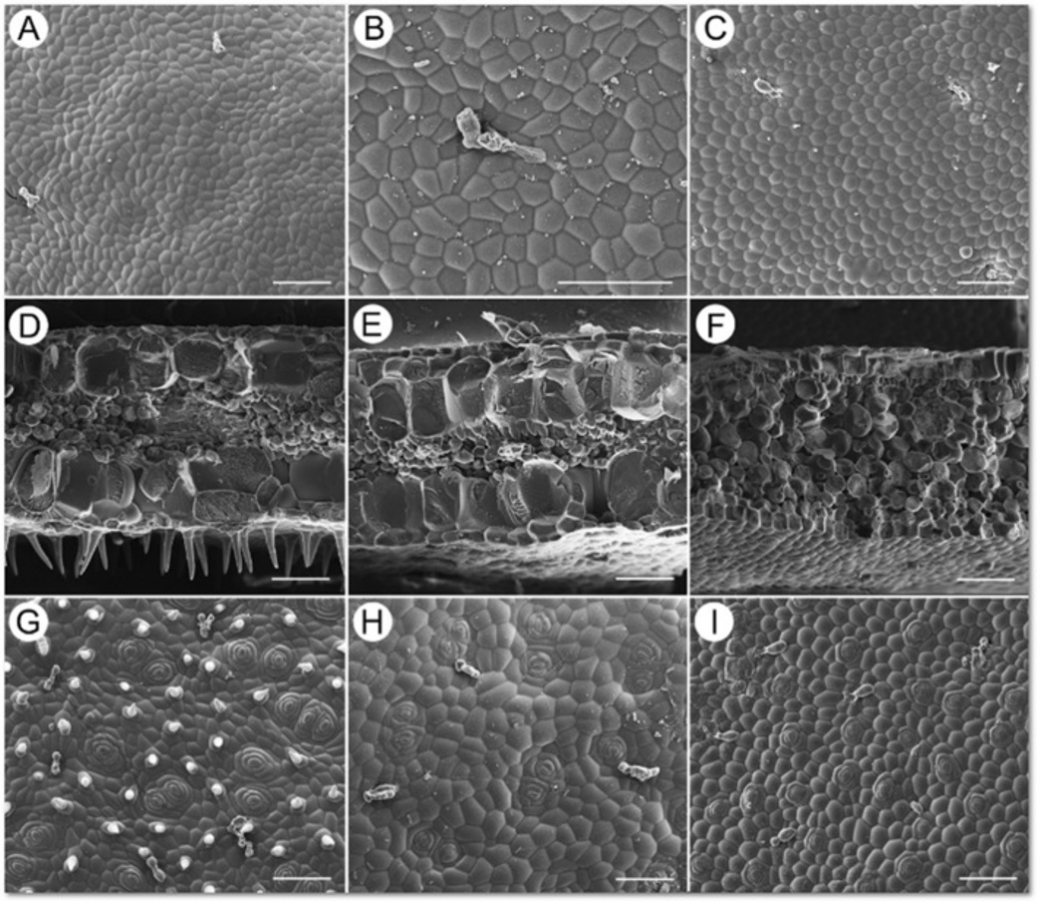
**Cryo SEM microphotographs of** ***Begonia*** **leaves. A**-**C**: Adaxial surface; **D**-**F**: Cross section; **G-I**: Abaxial surface; **A**, **D**, **G**: *Begonia guixiensis*; **B**, **E**, **H**: *B. cylindrica*; **C**, **F**, **I**: *B. longa*.

Adaxial surface with glandular trichomes (Figure 5A); ca. 700 μm thick (Figure 5D), epidermis biseriate, with a layer of thick hypodermis under both epidermis; abaxial surface with bicellular microtrichomes (conical-headed) and glandular trichomes (Figure 5D, G); stomata complex mostly clustered, helicocytic (Figure 5G).

#### Chromosome cytology

**Figure 6:**
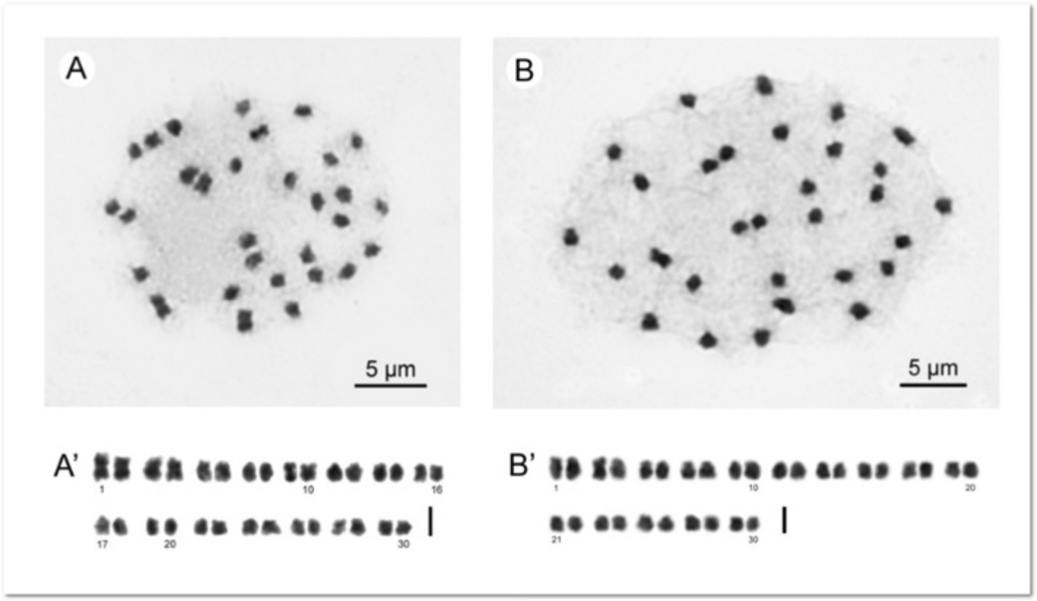
**Somatic chromosomes at metaphase of** ***Begonia guixiensis*** **and** ***B. longa*** **. A**, Microphotograph of *B. guixiensis* (*Peng et al. 20310,* 2*n* = 30); **B**, Microphotograph of *B. longa* (*Peng et al. 20076,* 2*n* = 30). **A’**, **B’**, Somatic chromosomes serially arranged by length and position of centromeres. Scale bars = 2 μm.

Somatic chromosomes at metaphase of *Begonia guixiensis* were determined to be 2*n* = 30 (Figure 6A).

The thirty chromosomes, ranging from ca. 1.1 to 2.1 μm long, showed a gradual change in chromosome length. Centromeres of most chromosomes are median or submedian. Satellite chromosomes were not observed.

#### Notes

**Figure 7:**
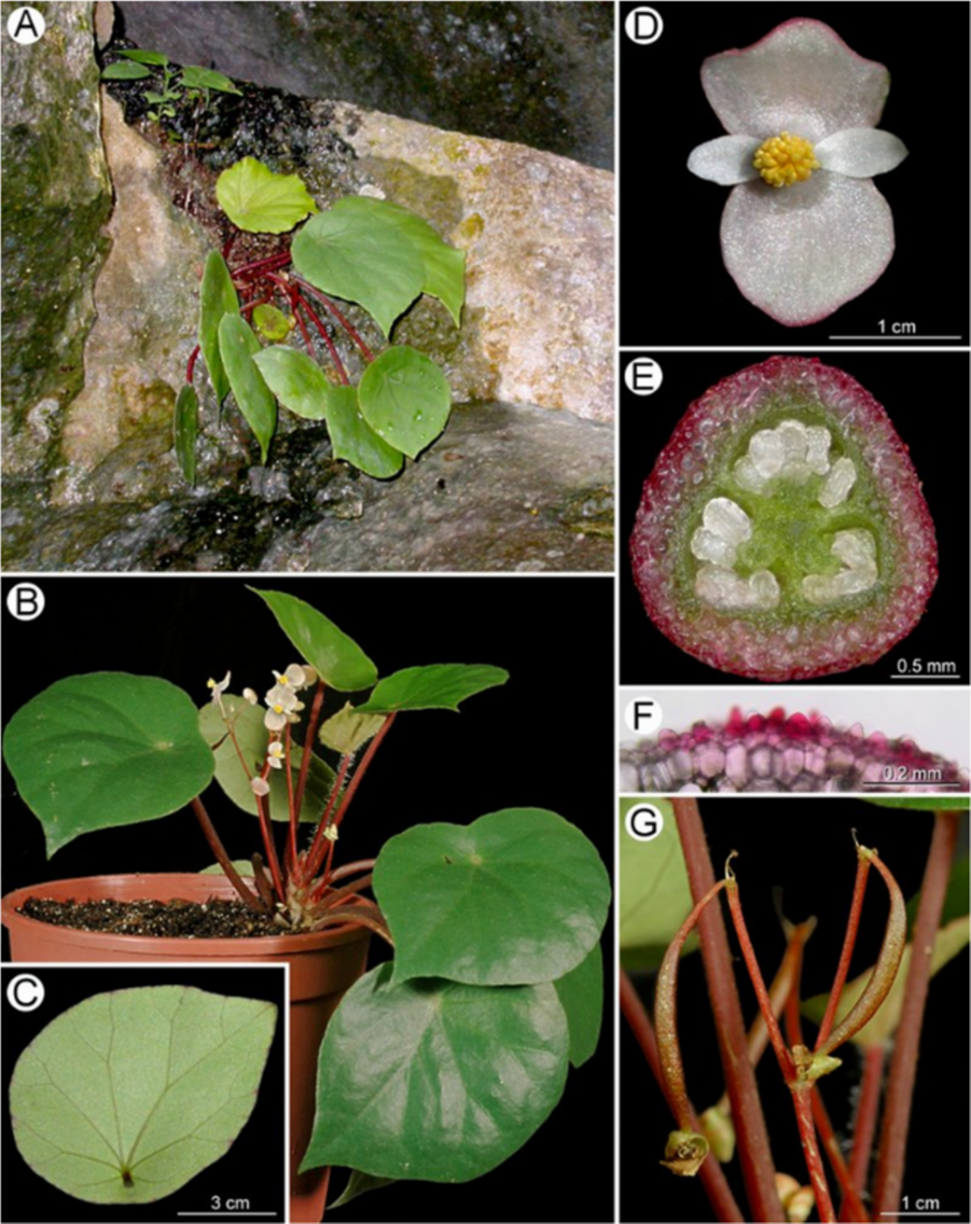
***Begonia cylindrica*** **D.R. Liang & X.X. Chen. A**, Habit and habitat; **B**, Habit at anthesis; **C**, Leaf, abaxial side; **D**, Staminate flower; **E**, Cross section of ovary; **F**, Unicellular microhairs of ovary (in LM); **G**, Narrowly cylindrical fruit with pendulous pedicel. A–G from *Leong et al.* 3635, D–F from *Leong et al.* 3371.

**Figure 8:**
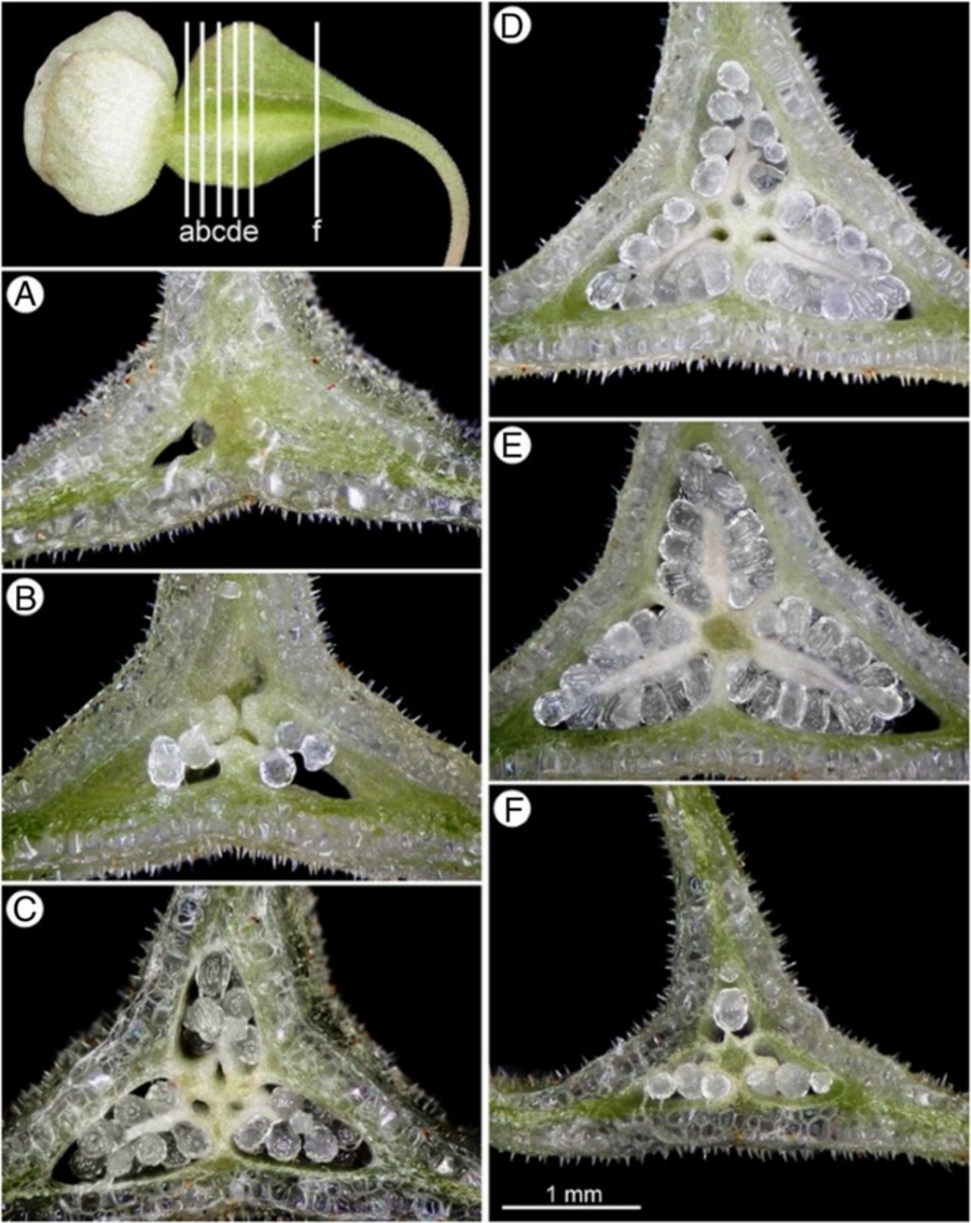
**Serial cross sections of an ovary of** ***Begonia guixiensis*** **Yan Liu, S.M. Ku & C.-I Peng. A**, The tip of cavity; **B**, Near the tip: three parietal placentae; **C**, Upper part: three placentae fused in the center but with three separate vascular bundles, forming gaps between placental branches; **D**, Upper-middle ovary: placental branches fusing on the sides; **E**, **F**, middle to lower ovary: vascular bundles united and assuming a 3-locular appearance. All from *Peng et al.* 20310.

*Begonia guixiensis* resembles *B. cylindrica* D.R. Liang and X.X. Chen (Figure 7) in the peltate, subcoriaceous leaves, differing by the shape of ovaries/fruits and placentation. Compared to the wingless and long-cylindric berrylike fruits of *B. cylindrica*, *B. guixiensis* possesses ovaries that are trigonous-ellipsoidal with three distinct wings. Its capsule-like fruits dry up and do not dehisce when mature. While the placentation of *B. cylindrica* is axile throughout the ovary, that of *B. guixiensis* is parietal on upper part of the ovary but the placental branches gradually fused side by side below and assuming a 3-locular appearance. Serial cross-sections to show the gradual changes are depicted in Figure 8.

Both *Begonia guixiensis* and *B. cylindrica* have clustered stomata and hypodermis (Figures D, E, G, H). These characters were also seen in *B. leprosa* (Peng et al. [2010]: figure nine-H, I), the most closely related species in the phylogeny (Figure 1), and are known in a number of limestone *Begonia* species from the Philippines and China (Hughes et al. [2011]; Rubite et al. [2014]).

*Begonia guixiensis* is also similar to *B. vietnamensis* H. Q. Nguyen and C.-I Peng (Nguyen et al., [2010]) in having peltate leaves, differing in the leaves uniformly green (vs. often mottled reddish brown between the veins) with petiole attachment manifestly displaced toward leaf base (vs. near 1/3 of blade length); petioles densely and minutely hispidulous (vs. glabrous); peduncles much shorter (2–6 cm vs. 28–32 cm long); carpellate flowers with 3 (vs. 2) tepals; ovary unilocular with parietal placentation near the summit but the placental branches gradually fused side by side below and assuming a 3-locular appearance (vs. 3-locular, placentae undivided); and the indehiscent (vs. dehiscent) fruit.

**Figure 9:**
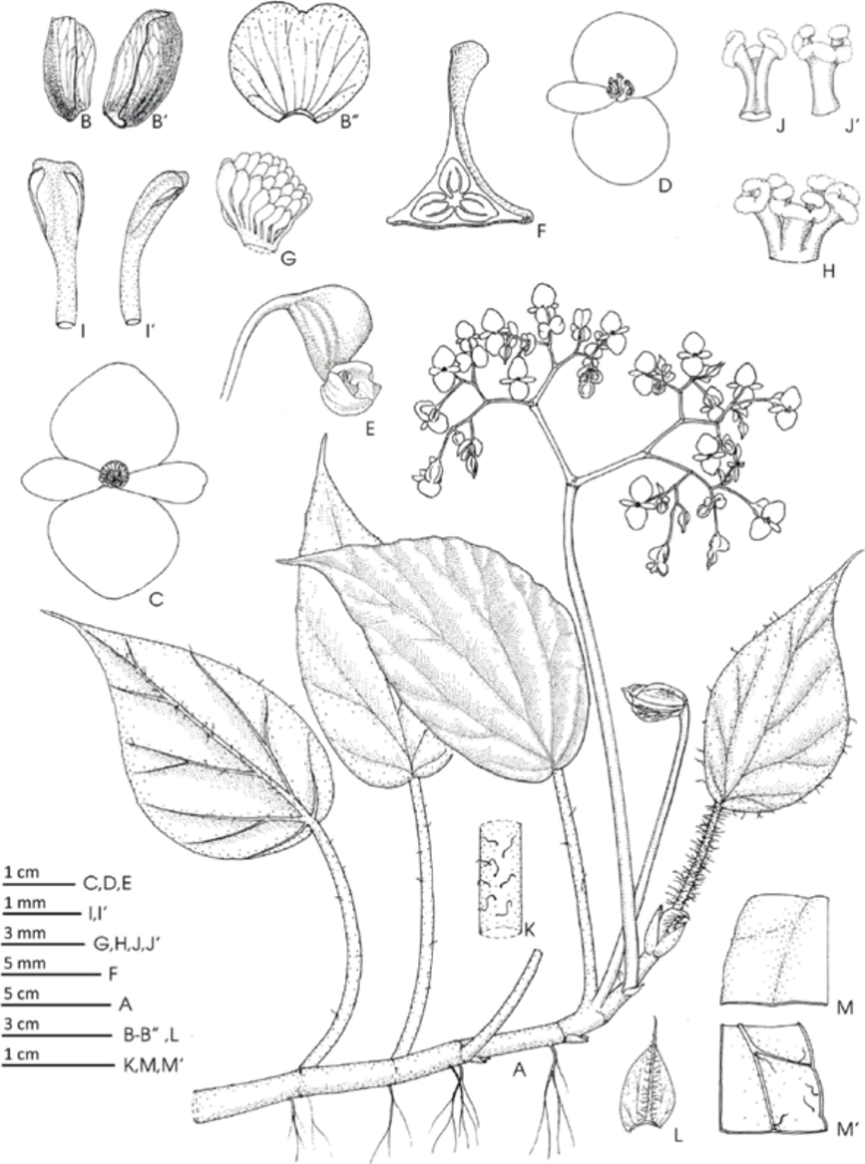
***Begonia longa*** **C.-I Peng & W.C. Leong. A**, Habit; **B**, **B**”, Bract; **C**, Staminate flower; **D**, Pistillate flower; **E**, Capsule; **F**, Cross section of ovary; **G**, Androecium; **H**, Styles; **I**, **I**’, Stamen; **J**, **J**’, Style, ventral and dorsal view; **K**, Portion of petiole; **L**, Stipule; **M**, **M**’, Portion of leaf, adaxial and abaxial side. All from C.-I *Peng et al. 20076*. Line drawing by Ya-Wen Hsueh.

**Figure 10:**
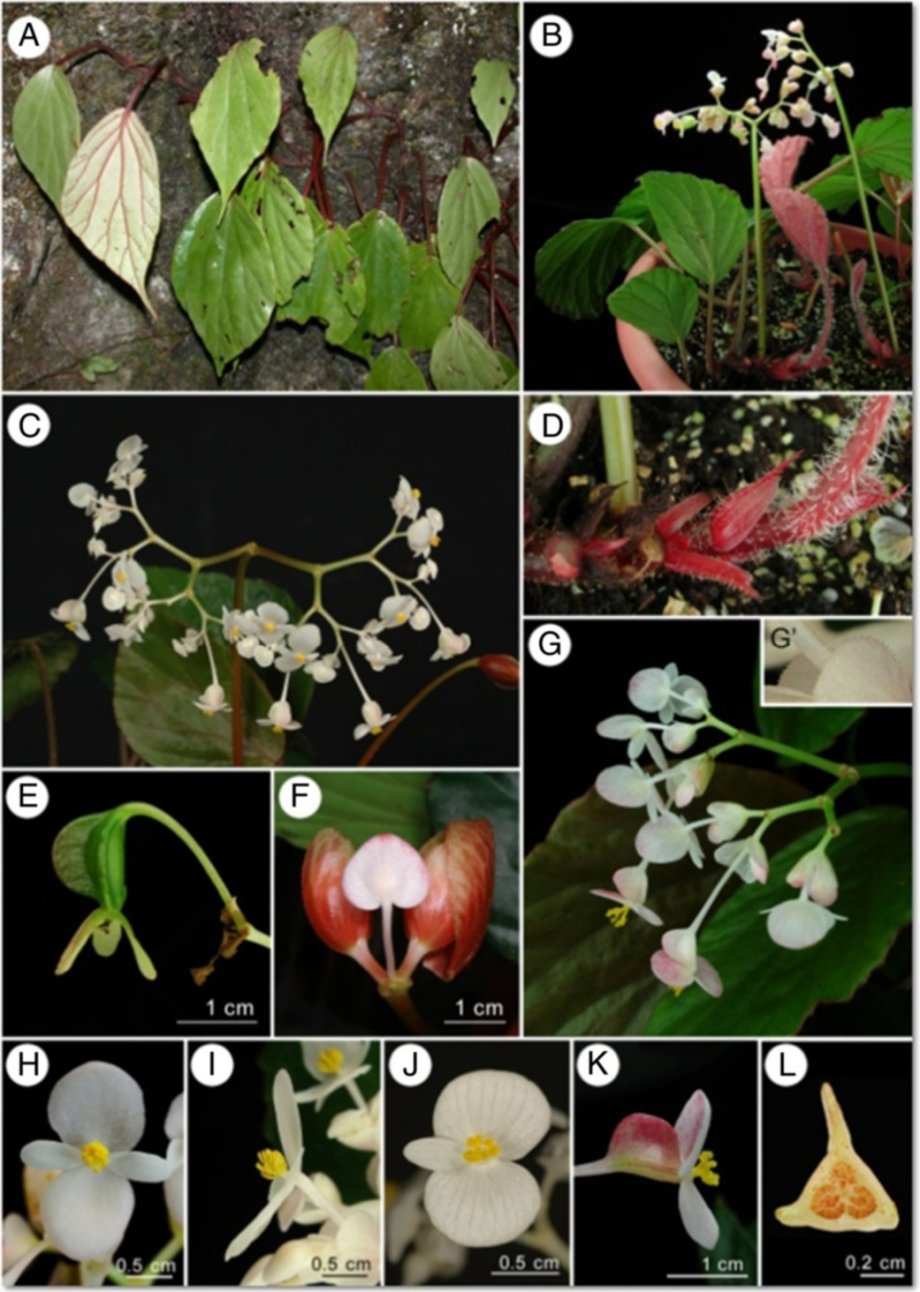
***Begonia longa*** **C.-I Peng & W.C. Leong. A**, Habit and habitat; **B**, Cultivated plant at anthesis; **C**, Inflorescence; **D**, Rhizome with stipules; **E**, Fruit with persistent tepals; **F**, Early stage of Inflorescence with bracts; **G**, Inflorescence; **G’**, Bract with glands on margin; **H**, Staminate flower, face view; **I**, Staminate flower, side view; **J**, Carpellate flower, face view; **K**, Carpellate flower, side view; **L**, Cross section of ovary. All from *C.-I Peng et al. 20076*.

2. ***Begonia longa*** C.-I Peng & W.C. Leong, sp. nov. (sect. *Coelocentrum*) —TYPE: VIETNAM. Ha Giang Province, Quan Ba District, Can Ty Community, Sin Suoi Ho Village, elev. 650–1500 m; on face of limestone rock, in shaded and moist broadleaf forest on mountain slope. Locally frequent, associated with *Hoya*, *Colocasia giganta*, *Asplenium*, *Amorphophallus*, *Pilea*, *Hemiboea* and *Nephrolepis cordifolia*. Living collection made on 12 Nov 2004; type specimens (in flower) pressed from plants cultivated in the experimental greenhouse, Academia Sinica, 10 May 2007. *Ching-I Peng*, *Wai-Chao Leong*, *Shin-Ming Ku*, *Tien Hiep Nguyen*, *Van The Pham & Xuan Tam Nguyen 20076-A* (holotype: HAST; isotypes: A, E, FRIM, MO, P). 長莖秋海棠 Figures 9 and 10.

Herbs monoecious, perennial, rhizomatous, lithophytic. *Rhizome* to 50 cm long, white villous when young; internodes 1–5 cm long, 0.5–0.8 cm across. *Stipules* thickly herbaceous, reddish, narrowly ovate to triangular, 1.7–2 cm long, 1–1.2 cm wide, margin entire, midrib prominently keeled into an arista 0.5 cm long, villous on midrib on the underside. *Leaves* alternate, basifixed, petioles reddish, (2–)5–14 cm long, 2–6 mm across, white villous when young, becoming brown tomentose and glabrescent when mature; leaf blade asymmetric, thickly herbaceous, dark green above, elliptic, (4–)6–16 cm long, (2.5–)3.5–8 cm wide, glabrous above, young leaves reddish, white villous along nerves beneath, base obtuse, margin entire to crenate, apex acuminate to caudate; venation pinnate or sub-palmate. *Inflorescences* axillary, arising directly from rhizome, dichasial, protandrous, 17–30 cm long, glabrous; peduncle well developed, 13.5–25 cm long, 2–5 mm across; bracts caducous, herbaceous, red, white or tinged pinkish, broadly elliptic or orbicular, boat-shaped, 1.8–2.6 cm long, 1.8–2.3 cm wide, glabrous, margin minutely glanduliferous. *Staminate flowers:* pedicel 0.8–1.8 cm long; tepals 4, white or tinged pinkish; outer two orbicular or broadly ovate, 0.8–1.4 cm long, 0.8–1.3 cm wide, glabrous, apex rounded; inner two narrowly elliptic to oblanceolate, 0.7–1.1 cm long, 0.3–0.4 cm wide; androecium zygomorphic, stamens 12–20, yellow, clavate; filaments unequal in length, 0.7–1.5 mm long, fused at base; anthers 1–1.5 mm long, apex retuse. *Carpellate flowers:* pedicel 1–3 cm long; tepals 3, white or tinged pinkish, persistent and greenish in fruit; outer two suborbicular, 0.9–1.3 cm long, 1.1–1.5 cm wide, apex rounded, inner one (or two) oblanceolate to obovate, 8–9 mm long, 2.5–5 mm wide; ovary white or greenish, sometimes tinged pinkish, glabrous, unequally 3-winged, 3-locular; placentation axile, bilamellate; styles 3, yellow, 2–3 mm long, nearly free; stigmas 2-cleft, in a spiralled band. *Capsules* nodding, unequally 3-winged; abaxial wing crescent-shaped, ca. 0.9–1.2 cm long, 4–5 mm tall, apex bluntly truncate, lateral wings much smaller, 2–3 mm long.

#### Ecology and distribution

*Begonia longa* is currently known only from Quan Ba District, Ha Giang Province, northern Vietnam (Figure 4). It grows on limestone rock face, in shaded and moist broadleaf forest. The plants were locally frequent, often associated with *Amorphophallus* sp., *Asplenium* sp., *Colocasia giganta*, *Hemiboea* sp.*, Hoya* sp., *Nephrolepis cordifolia and Pilea* sp.

#### Phenology

Flowering April–August; fruiting July–November.

#### Etymology

The specific epithet refers to the long creeping rhizome in this species.

#### Additional specimens examined

Vietnam. Ha Giang Province, Quan Ba (“Quang” Ba) District: Sin Suoi Ho Village and river; in degraded agricultural land among limestone rocks to ridge of exposed limestone under light canopy, 23° 06′ 57” N, 105° 01′ 47” E, elev. 514 m, N & S slopes. Occasional. 1 Apr, 2000. *D.K. Harder, N.T. Hiep, L.V. Averyanov, N.Q. Hieu & K. Daria 4868* (MO); Ha Giang Province, Quan Ba District: Can Ty Community, 23° 06′ 03” N, 105° 01′ 20” E, elev. 900 m, selectively logged forest on limestone, Bat Dai Son Protected Area, 9 Jul 2002. *H. Van der Werff, Nguyen Kim Dao, Bruce Gray, Do Tien Doan 17203* (HAST, MO); Ha Giang Province, Quan Ba District: Can Ty Community, Sin Suoi Ho Village, elev. 650–1500 m; on face of limestone rock, in shaded and moist broadleaf forest on mountain slope. Locally frequent.12 Nov 2004, *Ching-I Peng*, *Wai-Chao Leong*, *Shin-Ming Ku*, *Nguyen Tien Hiep*, *Pham Van The & Nguyen Xuan Tam 20076* (HAST);

#### Leaf anatomy and vestiture

Adaxial surface with glandular trichomes (Figure 5C); cross section ca. 700 μm thick (Figure 5F), epidermis single-layered, hypodermis absent; abaxial surface with glandular trichomes (Figure 5I); stomata complex single, helicocytic (Figure 5I).

#### Chromosome cytology

Somatic chromosomes at metaphase of *Begonia longa* were determined to be 2*n* = 30 (Figure 6B). The thirty chromosomes gradually varied from ca. 1.2 to 1.8 μm long in length. Most chromosomes have centromeres at median, submedian and subterminal positions. Satellites were not observed.

The Asian section *Coelocentrum* consistently shows the chromosome number of 2*n* = 30, with the exception of some probable autotriploid individuals with 2*n* = 45 in *B. longgangensis* (Peng et al. [2013]). Chromosome numbers of the two new species, *B. guixiensis* (see above) and *B. longa*, studied here are in agreement with previous reports for species of *Begonia* in this section.

#### Notes

In aspect, *B. longa* resembles *B. brevipedunculata* Y.M. Shui (Shui [2006]) in the leaf shape, differing by many features such as the elongate internodes, shorter petioles, smaller leaves, longer peduncles and its 3-locular ovary. Detailed comparison of salient features of the two species is provided in Table 1.

**Table 1:** **Comparison of** ***Begonia longa*** **C.-I Peng & W.C. Leong and** ***B. brevipedunculata*** **Y.M. Shui**

	***Begonia longa***	***Begonia brevipedunculata***
Rhizome and habit	Elongate to 50 cm long, internodes 1–5 cm long	Short, ca. 2.5 cm long, leaves caespitose
Petioles	(2–)5–14 cm long	17–24 cm long
Leaves	(4–)6–16 cm long, (2.5–)3.5–8 cm wide	15–19 cm long, 7–10 cm wide
Indumentum on leaf abaxial surface	Villous along veins	Densely clothed with clavate glandular hairs
Peduncle	13-25 cm long	Very short, 2–4 cm long
Ovarian locules	3	2

## Conclusions

A careful study of the literature, herbarium specimens and living plants, both in the wild and in cultivation in the experimental greenhouse, supports the recognition of the two new species.

## Authors’ original submitted files for images

Below are the links to the authors’ original submitted files for images. Authors’ original file for figure 1 Authors’ original file for figure 2 Authors’ original file for figure 3 Authors’ original file for figure 4 Authors’ original file for figure 5 Authors’ original file for figure 6 Authors’ original file for figure 7 Authors’ original file for figure 8 Authors’ original file for figure 9 Authors’ original file for figure 10

## References

[CR1] Averyanov LV, Nguyen HQ (2012). Eleven new species of *Begonia* L. (Begoniaceae) from Laos and Vietnam. Turczaninowia.

[CR2] Chung K-F, Huang H-Y, Peng C-I, Xu W-B (2013). *Primulina mabaensis* (Gesneriaceae), a new species from a limestone cave of northern Guangdong, China. Phytotaxa.

[CR3] Chung K-F, Leong W-C, Rubite RR, Repin R, Kiew R, Liu Y, Peng C-I (2014). Phylogenetic analyses of *Begonia* sect. *Coelocentrum* and allied limestone species of China shed light on the evolution of Sino-Vietnamese karst flora. Bot Stud.

[CR4] Clements R, Sodhi NS, Schilthuizen M, Ng PKL (2006). Limestone karsts of Southeast Asia: imperiled arks of biodiversity. Bioscience.

[CR5] Doorenbos J, Sosef MSM, de Wilde JJFE (1998). The Sections of Begonia.

[CR6] Gu C-Z, Peng C-I, Turland NJ, Wu ZY, Raven PH, Hong DY (2007). Begoniaceae. Flora of China.

[CR7] Hou M-F, López-Pujol J, Qin H-N, Wang L-S, Liu Y (2010). Distribution pattern and conservation priorities for vascular plants in Southern China: Guangxi Province as a case study. Bot Stud.

[CR8] Huang Y-S, Xu W-B, Peng R-C, Liu Y (2011). A new variety of *Hemiboea* (Gesneriaceae) from limestone areas in Guangxi, China. Taiwania.

[CR9] Hughes M, Rubite RR, Kono Y, Peng C-I (2011). *Begonia blancii* (sect. *Diploclinium*, Begoniaceae), a new species endemic to the Philippine island of Palawan. Bot Stud.

[CR10] Ku S-M, Kono Y, Liu Y (2008). *Begonia pengii* (sect. *Coelocentrum*, Begoniaceae), a new species from limestone areas in Guangxi, China. Bot Stud.

[CR11] Ku T-C, Ku T-C (1999). Begoniaceae. Flora Reipublicae Popularis Sinicae, vol. 52(1).

[CR12] Levan A, Fredga K, Sandberg AA (1964). Nomenclature for centromeric position on chromosomes. Hereditas.

[CR13] Lin C-R, Liu Y, Nong D-X, Kono Y, Peng C-I (2013). *Aspidistra crassifila* (Asparagaceae), a new species from Guangxi, China. Bot Stud.

[CR14] Liu Y, Ku S-M, Peng C-I (2007). *Begonia bamaensis* (sect. *Coelocentrum*, Begoniaceae), a new species from limestone areas in Guangxi, China. Bot Stud.

[CR15] Nguyen QH, Peng C-I, Ku S-M (2010). *Begonia vietnamensis*, an attractive new species with peltate leaves from Vietnam. Begonian.

[CR16] Peng C-I, Hsieh T-Y, Nguyen QH (2007). *Begonia kui* (sect. *Coelocentrum*, Begoniaceae), a new species from Vietnam. Bot Stud.

[CR17] Peng C-I, Liu Y, Ku S-M (2008). *Begonia aurantiflora* (sect. *Coelocentrum*, Begoniaceae), a new species from limestone areas in Guangxi, China. Bot Stud.

[CR18] Peng C-I, Liu Y, Ku S-M, Kono Y, Chung K-F (2010). *Begonia* × *breviscapa* (Begoniaceae), a new intersectional natural hybrid from limestone areas in Guangxi, China. Bot Stud.

[CR19] Peng C-I, Ku S-M, Kono Y, Liu Y (2012). *Begonia chongzuoensis* (sect. *Coelocentrum*, Begoniaceae), a new calciphile from Guangxi, China. Bot Stud.

[CR20] Peng C-I, Yang H-A, Kono Y, Chung K-F, Huang Y-S, Wu W-H, Liu Y (2013). Novelties in *Begonia* sect. *Coelocentrum*: *B. longgangensis* and *B. ferox* from limestone areas in Guangxi, China. Bot Stud.

[CR21] Peng C-I, Jin X-H, Ku S-M, Kono Y, Huang H-Y, Yang H-A (2014a). *Begonia wuzhishanensis* (sect. *Diploclinium*, Begoniaceae), a new species from Hainan Island, China. Bot Stud.

[CR22] Peng C-I, Wang H, Kono Y, Yang H-A (2014b). *Begonia wui-senioris* (sect. *Platycentrum*, Begoniaceae), a new species from Myanmar. Bot Stud.

[CR23] Rubite RR, Callado JR, Kono Y, Yang H-A (2014). *Begonia chingipengii* (sect. *Baryandra*, Begoniaceae), a new species from Luzon Island, Philippines. Phytotaxa.

[CR24] Shui Y-M (2006). A new species of *Begonia* section *Platycentrum* (Begoniaceae) from Vietnam. Novon.

[CR25] Tebbitt MC, Lowe-Forrest L, Santoriello A, Clement WL, Swensen SM (2006). Phylogenetic relationships of Asian *Begonia*, with an emphasis on the evolution of rain-ballist and animal dispersal mechanisms in sections *Platycentrum*, *Sphenanthera* and *Leprosae*. Syst Bot.

[CR26] Thomas DC, Hughes M, Phutthai T, Rajbhandary S, Rubite R, Ardi WH, Richardson JE (2011). A non-coding plastid DNA phylogeny of Asian *Begonia* (Begoniaceae): evidence for morphological homoplasy and sectional polyphyly. Mol Phylogenet Evol.

[CR27] Wei Y-G, Monro AK, Wang W-T (2013). Additions to the Flora of China: three new species of *Elatostema* (Urticaceae) from Guangxi. Phytotaxa.

[CR28] Xu W-B, Pan B, Liu Y (2011). *Petrocosmea huanjiangensis*, a new species of Gesneriaceae from limestone areas in Guangxi, China. Novon.

[CR29] Xu W-B, Pan B, Liu Y, Peng C-I, Chung K-F (2012). Two new species, *Primulina multifida* and *P. pseudomollifolia* (Gesneriaceae), from karst caves in Guangxi, China. Bot Stud.

[CR30] Xu W-B, Zhang Q, Wen F, Liao W-B, Pan B, Chang H, Chung K-F (2012). Nine new combinations and one new name of *Primulina* (Gesneriaceae) from South China. Phytotaxa.

[CR31] Xu W-B, Meng T, Zhang Q, Wu W-H, Liu Y, Chung K-F (2014). *Petrocodon* (Gesneriaceae) in the limestone karsts of Guangxi, China: three new species and a new combination based on morphological and molecular evidence. Syst Bot.

[CR32] Xu Z-R (1995). A study of the vegetation and floristic affinity of the limestone forests in southern and southwestern China. Ann Missouri Bot Gard.

[CR33] Zhang L-R, Zhang Z-X, Meng R, Yu S-X (2014). *Impatiens pterocaulis* sp. nov. (Balsaminaceae) from Guangxi, China. Nord J Bot.

