# Supplementary figures and images for: Two new species of Begonia sect. Coelocentrum, B. guixiensis and B. longa, from Sino-Vietnamese limestone karsts
Source: Bot Stud. 2014 Jun 28;55:52. doi: 10.1186/s40529-014-0052-8 (PMC5430358; doi:10.1186/s40529-014-0052-8)

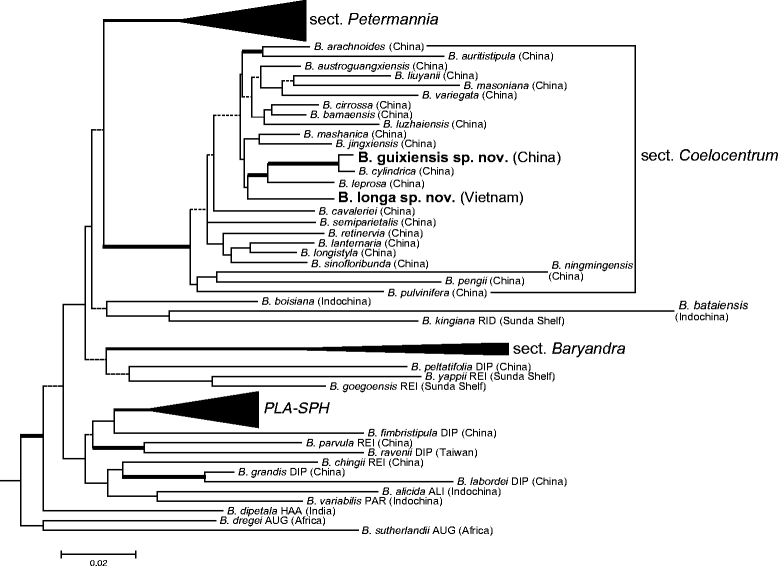

Supplement: Supplementary file 1 — Authors’ original file for figure 1 [file 40529_2014_52_MOESM1_ESM.gif]

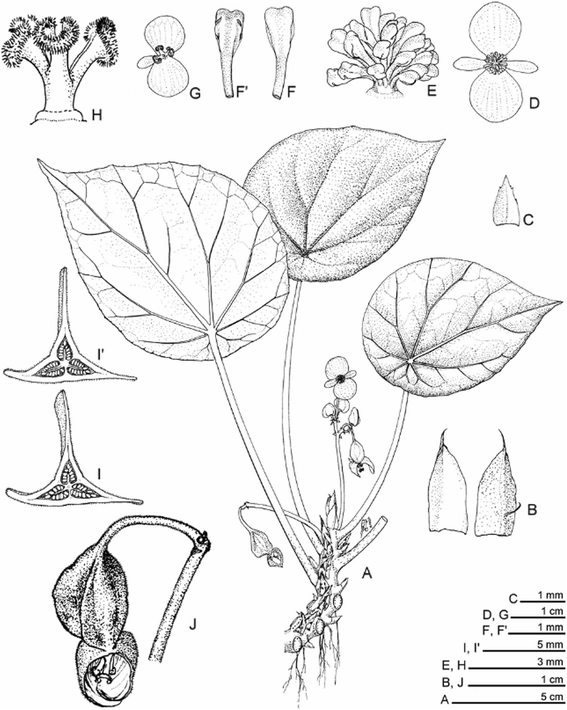

Supplement: Supplementary file 2 — Authors’ original file for figure 2 [file 40529_2014_52_MOESM2_ESM.gif]

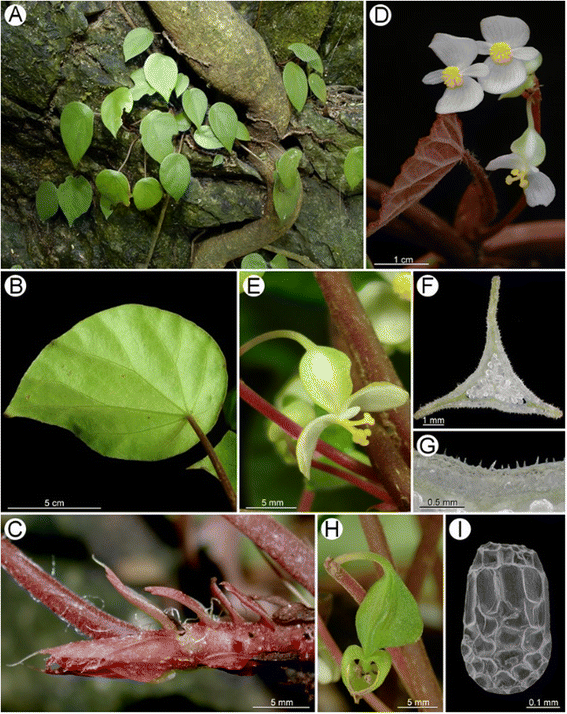

Supplement: Supplementary file 3 — Authors’ original file for figure 3 [file 40529_2014_52_MOESM3_ESM.gif]

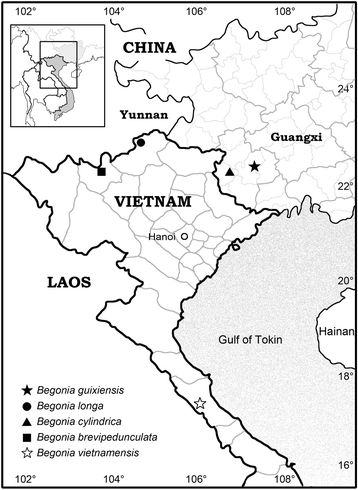

Supplement: Supplementary file 4 — Authors’ original file for figure 4 [file 40529_2014_52_MOESM4_ESM.gif]

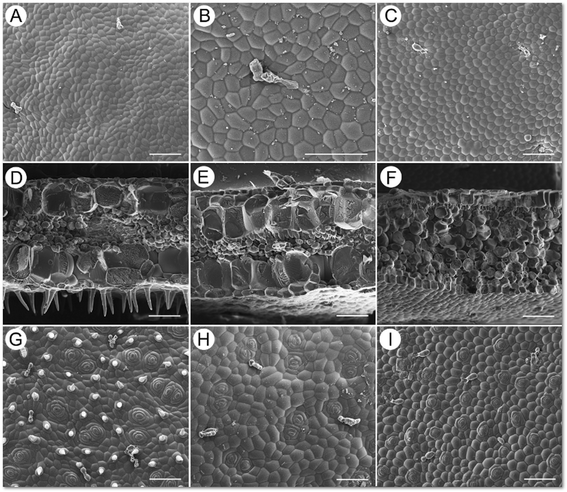

Supplement: Supplementary file 5 — Authors’ original file for figure 5 [file 40529_2014_52_MOESM5_ESM.gif]

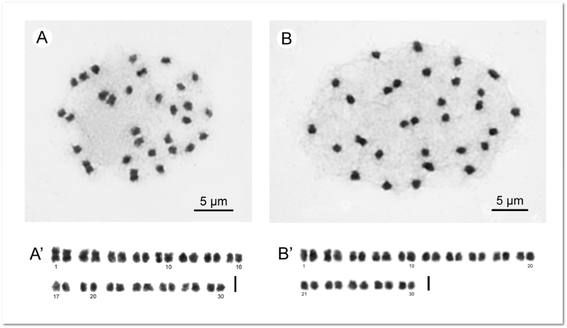

Supplement: Supplementary file 6 — Authors’ original file for figure 6 [file 40529_2014_52_MOESM6_ESM.gif]

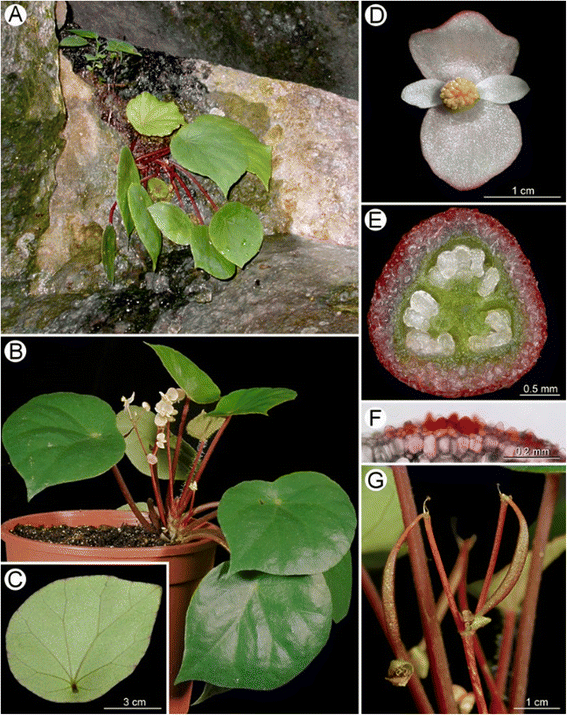

Supplement: Supplementary file 7 — Authors’ original file for figure 7 [file 40529_2014_52_MOESM7_ESM.gif]

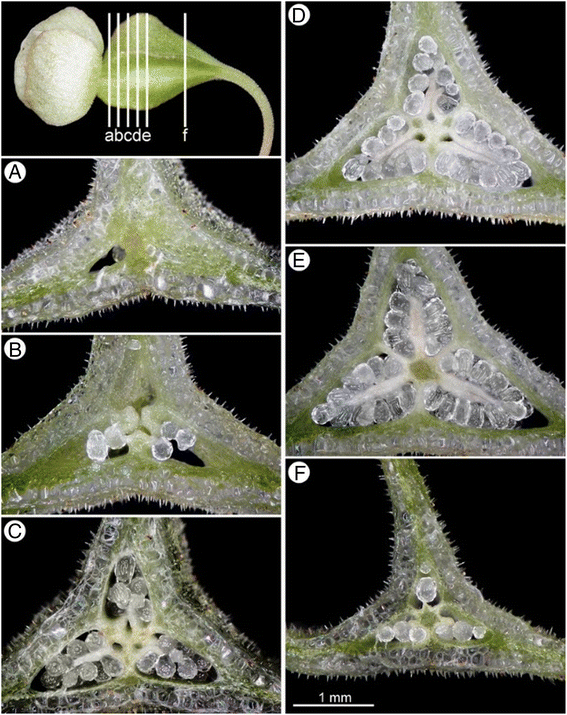

Supplement: Supplementary file 8 — Authors’ original file for figure 8 [file 40529_2014_52_MOESM8_ESM.gif]

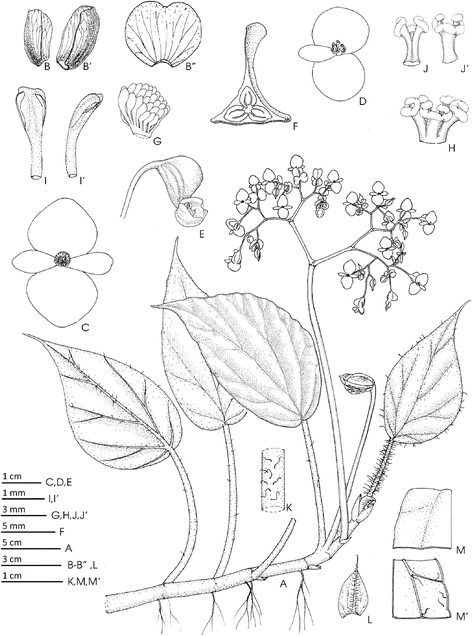

Supplement: Supplementary file 9 — Authors’ original file for figure 9 [file 40529_2014_52_MOESM9_ESM.gif]

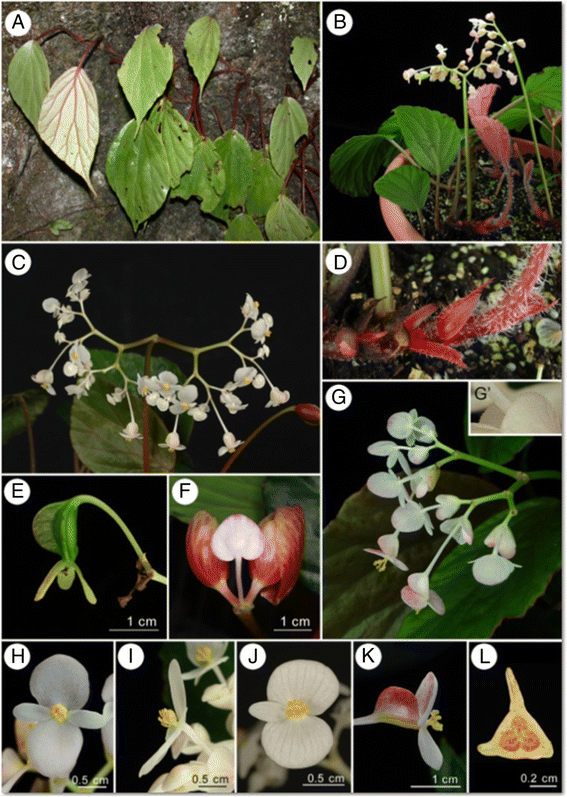

Supplement: Supplementary file 10 — Authors’ original file for figure 10 [file 40529_2014_52_MOESM10_ESM.gif]
